# X-ray optics for advanced ultrafast pump–probe X-ray experiments at SACLA^1^

**DOI:** 10.1107/S1600577518018362

**Published:** 2019-02-22

**Authors:** Tetsuo Katayama, Takashi Hirano, Yuki Morioka, Yasuhisa Sano, Taito Osaka, Shigeki Owada, Tadashi Togashi, Makina Yabashi

**Affiliations:** a XFEL Division, Japan Synchrotron Radiation Research Institute, 1-1-1 Kouto, Sayo-cho, Sayo-gun, Hyogo 679-5148, Japan; b RIKEN SPring-8 Center, 1-1-1 Kouto, Sayo-cho, Sayo-gun, Hyogo 679-5148, Japan; c Department of Precision Science and Technology, Graduate School of Engineering, Osaka University, 2-1 Yamada-oka, Suita, Osaka 565-0871, Japan

**Keywords:** X-ray free electron laser, X-ray optics, pump–probe X-ray measurements

## Abstract

X-ray optics were implemented for advanced ultrafast X-ray experiments with different techniques at the hard X-ray beamline BL3 of SPring-8 Ångstrom Compact free-electron LAser. A double channel-cut crystal monochromator (DCCM) and compound refractive lenses (CRLs) were installed to tailor the beam conditions. These X-ray optics can work simultaneously with an arrival-timing monitor that compensates for timing jitter and drift. Inner-walls of channel-cut crystals (CCs) in the DCCM were processed by plasma chemical vaporization machining to remove crystallographic damage. Four-bounced reflection profiles of the CCs were investigated and excellent diffraction qualities were achieved. The use of CRLs enabled two-dimensional X-ray focusing with a spot size of ∼1.5 µm × 1.5 µm full width at half-maximum, while keeping reasonable throughputs for a wide photon energy range of 5−15 keV.

## Abstract 2

A double channel-cut crystal monochromator and compound refractive lenses were implemented for advanced pump–probe X-ray experiments at SACLA BL3.

## 1. Introduction   

At SPring-8 Ångstom Compact free-electron LAser (SACLA; Ishikawa *et al.*, 2012 ▸), constructed in Harima, Japan, two hard X-ray beamlines (BL2 and BL3; Tono *et al.*, 2013 ▸) and one soft X-ray beamline (BL1; Owada *et al.*, 2018 ▸) are in operation for users, providing brilliant, ultrafast X-ray free-electron laser (XFEL) pulses. For conducting pump–probe experiments combining hard X-rays and synchronized optical laser pulses, experimental hutch 2 (EH2) of BL3 is widely utilized. One of the advantages of using EH2 for pump–probe experiments is the proximity to an arrival-timing monitor (Katayama *et al.*, 2016 ▸), which is located in experimental hutch 1 (EH1) of BL3. With this diagnostics, one is able to measure relative arrival timings between X-ray and optical pulses simultaneously with experiments, to compensate them with a post-process analysis and improve the time resolution to the ∼10 fs level.

The arrival-timing monitor is based on a beam-branching scheme using a transmission grating (Katayama *et al.*, 2013 ▸, 2016 ▸; David *et al.*, 2015 ▸; Makita *et al.*, 2015 ▸), which splits an incoming ‘pink’ X-ray beam with a bandwidth of 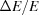 ≃ 5 × 10^−3^ into a main branch and sub-branches. One of the sub-branches (−1st-order) dedicated for the arrival-timing diagnostics is guided to a GaAs thin crystal to produce a transient change of optical transmittance (Sato *et al.*, 2015 ▸). This modulation is probed with an overall accuracy of 7.0 fs with a spatial encoding technique. The arrival-timing data can be conveniently extracted with the software package *Timing Monitor Analyzer* (*TMA*; Nakajima *et al.*, 2018 ▸) in real time. On the other hand, the main (0th-order transmission) branch keeping most of the original intensity is provided for pump–probe experiments in EH2. For accommodating diverse applications with complementary X-ray techniques, it is highly preferable to tailor the beam conditions, *i.e.* bandwidth and beam size, of the 0th-order transmission branch without preventing the arrival-timing diagnostics. For example, the capability to reduce the bandwidth from the original value to 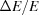 ≃ 1 × 10^−4^ with a monochromator will enable users to swiftly switch between time-resolved X-ray emission spectroscopy (TR-XES; Alonso-Mori *et al.*, 2016 ▸) and absorption spectroscopy (TR-XAS; Obara *et al.*, 2017 ▸; Uemura *et al.*, 2016 ▸; Ogi *et al.*, 2015 ▸), even during their beam time. In this paper, we describe a double channel-cut crystal monochromator (DCCM) and compound refractive lenses (CRLs; Lengeler *et al.*, 1999 ▸; Snigirev *et al.*, 1996 ▸), implemented for extending the XFEL scientific capabilities at BL3 of SACLA. These X-ray optical devices were carefully designed to avoid interference with the −1st-order branch for the arrival-timing diagnostics. We report the results of the performance test using the XFEL beam.

## 2. Design concept   

### 2.1. Double channel-cut crystal monochromator   

**Figure 1:**
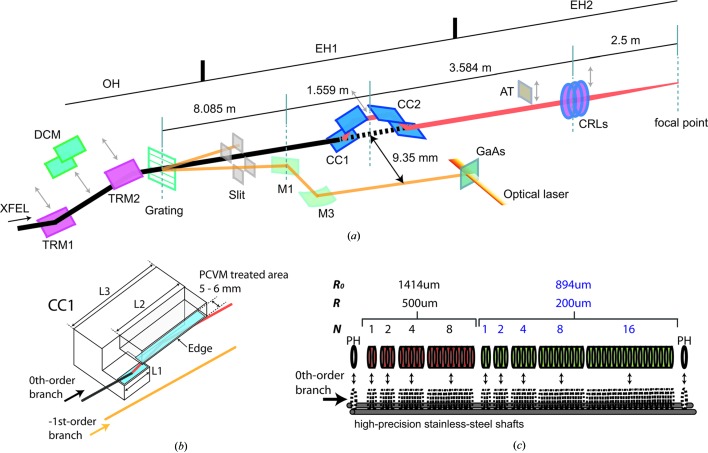
(*a*) Overview of the X-ray optical system for pump–probe experiments at BL3 of SACLA. TRM1 and TRM2: total reflection mirrors for transporting the XFEL beam with the full bandwidth; DCM: double-crystal monochromator to transport the XFEL beam with limited bandwidth of the order of 10^−4^; Grating: diffraction grating to split the incident beam into multiple branches; M1 and M3: mirrors to reflect the −1st-order branch for the arrival-timing diagnostics; CC1 and CC2: first and second channel-cut crystals of the DCCM; AT: silicon attenuators; CRLs: compound refractive lenses implemented in EH2. Dimensions of CC1 and CC2 are in mirror symmetry. (*b*) Schematic of CC1. The PCVM-treated areas of the inner-wall surfaces are indicated as blue rectangles. Parameters of L1, L2 and L3 are listed in Table 1 ▸. (*c*) Schematic of the CRLs. *R*_0_, *R* and *N* are the nominal aperture, the radius of curvature at the apex and the number of lenses in the stacks, respectively. PH is a pinhole with an aperture of 1.0 mm.

The DCCM consists of a pair of channel-cut crystals (CCs) with a (+, −, −, +) geometry to keep the beam axis unchanged even when the monochromator is utilized, as shown in Fig. 1 ▸(*a*). The design parameters of the CCs are listed in Table 1 ▸. Two types of CCs, Type *A* and Type *B*, were prepared to cover a wide photon energy range; the Type *A* CCs were used in the present study. With the DCCM, the four-bounced reflection gives an extra delay for the X-ray path with respect to the straight trajectory, which changes the relative arrival timing between X-ray and optical pulses. This timing offset Δ*t* caused by the DCCM (Hirano *et al.*, 2016 ▸) can be written as
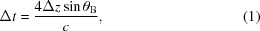
where Δ*z*, θ_B_ and *c* are the gap of the CCs, the Bragg angle and the speed of light, respectively. Compensation of 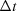 is simply achieved by using an optical delay stage and an electric delay unit for the optical laser.

The DCCM was installed at a distance of 1.559 m from a flat mirror (M1) that reflected only the −1st-order branch to yield a horizontal separation of 9.35 mm from the 0th-order branch [Fig. 1 ▸(*a*)]. Owing to this separation, the 0th-order branch is monochromatized by using 1–8 mm areas from the edges of the CCs. The effective inner walls of the CCs [Fig. 1 ▸(*b*)] were processed by plasma chemical vaporization machining (PCVM; Hirano *et al.*, 2016 ▸; Osaka *et al.*, 2012 ▸, 2013 ▸; Mori *et al.*, 2000 ▸), an etching method using atmospheric-pressure plasma. By applying the PCVM treatment, we were able to eliminate subsurface crystallographic damage, while maintaining a smooth surface. The use of such damage-free optics is important for coherent applications that require a speckle-free XFEL beam without a wavefront distortion.

### 2.2. Compound refractive lenses   

In EH2, the size of the unfocused XFEL beam is typically 350 µm × 350 µm full width at half-maximum (FWHM) at 10 keV. Beryllium CRLs (RXOPTICS) consisting of two concave parabolic surfaces were employed to control the beam size of the 0th-order branch by two-dimensional focusing. The CRL assembly containing nine lenses stacks and two pinholes [see Fig. 1 ▸(*c*)] was installed at a distance of 2.5 m from the sample position in EH2. Nominal apertures 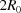 (the radius of curvature at the apex *R*) of the lenses are 1414 µm (500 µm) for the first four stacks and 894 µm (200 µm) for the next five stacks. Pneumatic actuators move these stacks and pinholes independently, to change the number of lenses. The tunability of the lens combination allows microfocusing at the sample position in a wide photon energy range of 5–15 keV. When inserted, the stacks are mounted on two parallel high-precision shafts by a tension spring so as to align the optical axes of lenses. The whole assembly is routinely adjusted in orientations and translations to center the optical axes on the 0th-order branch.

## 3. Performance   

### 3.1. Spatial profile and throughput of the DCCM   

**Figure 2:**
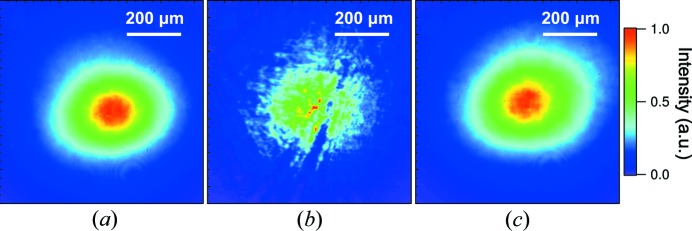
Averaged spatial beam profiles of ∼150 XFEL pulses. (*a*) Incident monochromatic beam through DCM. Four-bounced reflected beams from a pair of Type *A* CCs with (*b*) PCVM-untreated areas and (*c*) PCVM-treated areas.

**Figure 3:**
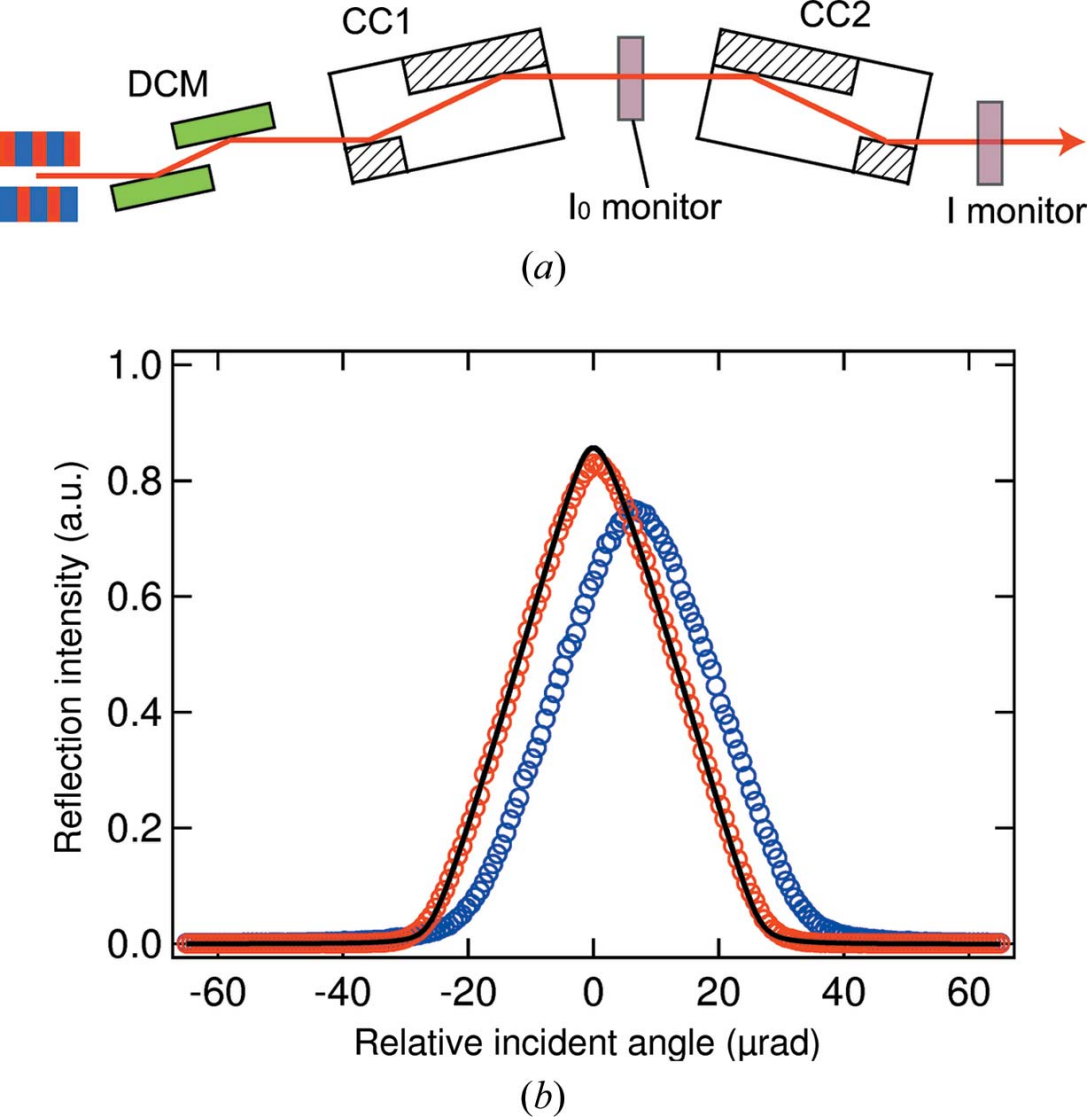
(*a*) Schematic diagram for the rocking curve measurements. The shot-to-shot pulse energies were recorded with transmissive beam intensity monitors (*I*_0_ and *I* monitors) consisting of a 15 µm nanocrystal diamond film and Si photodiodes (Hamamatsu, S3590-09). (*b*) Rocking curve measured with a rotation of CC2 at 10 keV. The red and blue circles correspond to experimental data measured at the PCVM-treated parts and the untreated parts, respectively. For each data point, approximately 300 XFEL pulses were used. The black line is the calculated curve, assuming a beam divergence of 2 µrad.

We investigated the performance of a pair of Type *A* CCs using 10 keV monochromatic XFEL pulses through a Si(111) double-crystal monochromator (DCM) in an optics hutch (OH). An averaged spatial profile of the incident beam without CCs is displayed in Fig. 2 ▸(*a*), which was recorded with a complementary metal oxide semiconductor (CMOS) camera (Hamamatsu, ORCA-Flash4.0, effective pixel size of 0.63 µm) placed at a sample position of EH2. Figs. 2 ▸(*b*) and 2(*c*) show averaged profiles with four-bounced Si(111) reflections of a pair of CCs. A number of speckles and scratches originating from crystallographic damage were found in the profile reflected on the areas untreated with PCVM [Fig. 2 ▸(*b*)]. In contrast, on the areas to be treated, we obtained an excellent reflection profile with little damage-induced speckles as shown in Fig. 2 ▸(*c*), which was similar to that of the incident beam. Fig. 3 ▸ shows the rocking curves with a rotation of the second CC. The experimental result measured at the PCVM-treated parts agreed with the calculation using the dynamical theory of X-ray diffraction, while the curve at the untreated parts exhibited a shift, broadening and weakening of the reflection peak originating from residual crystallographic damage and strain. The measured peak reflectivities are 83% and 75% at the PCVM-treated and untreated parts, respectively. These observations unambiguously indicate that the diffraction qualities of CCs were improved by removing crystallographic damage via PCVM.

### 3.2. Focusing property of CRLs   

**Figure 4:**
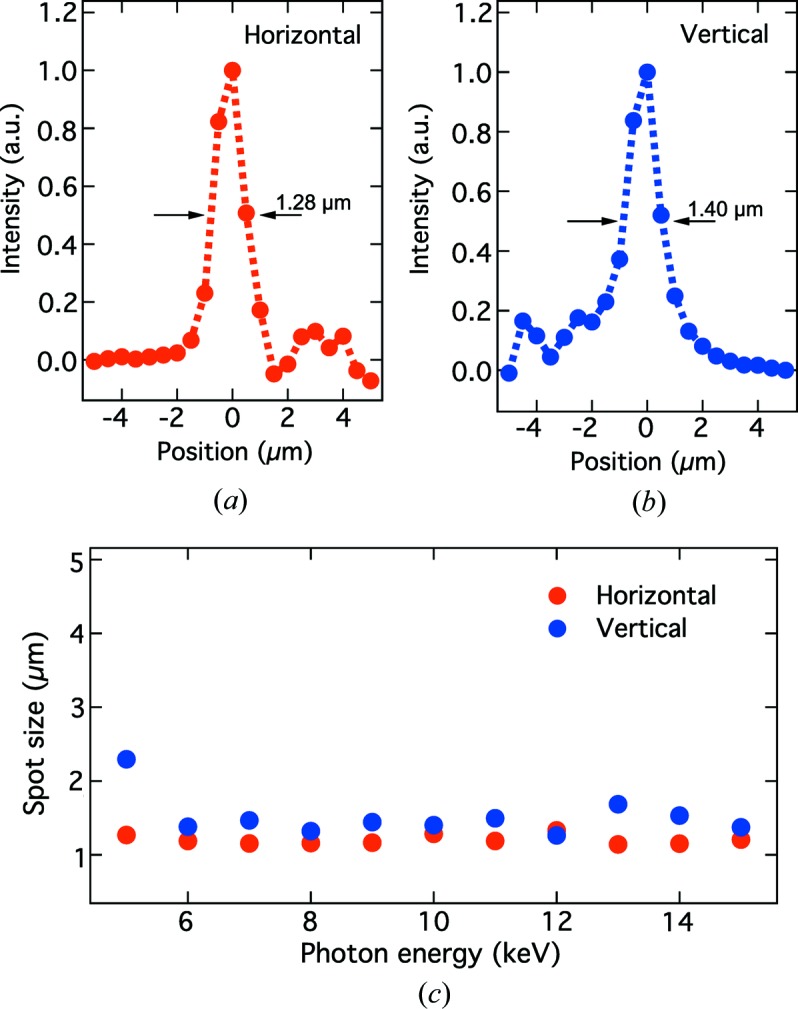
(*a*), (*b*) Intensity distributions of the focused beam at 10 keV, measured using the knife-edge scanning method. (*c*) The FWHM spot size at the sample position as a function of the photon energy. The parameters of the CRLs are presented in Table 2 ▸.

We tested two-dimensional focusing of CRLs using the XFEL beam with the full bandwidth through double total reflection mirrors (TRM1 and TRM2) in OH. The focused beam size at the sample position in EH2 was characterized with a knife-edge scanning method using an Au wire (200 µm diameter). The applied CRL parameters are listed in Table 2 ▸. Fig. 4 ▸ shows intensity profiles achieved for the focused beam with a photon energy of 10 keV. The measured spot size was 1.28 µm FWHM in the horizontal direction [Fig. 4 ▸(*a*)] and 1.40 µm FWHM in the vertical direction [Fig. 4 ▸(*b*)]. Similar results were obtained at various photon energies within the range 5–15 keV, as shown in Fig. 4 ▸(*c*).

**Figure 5:**
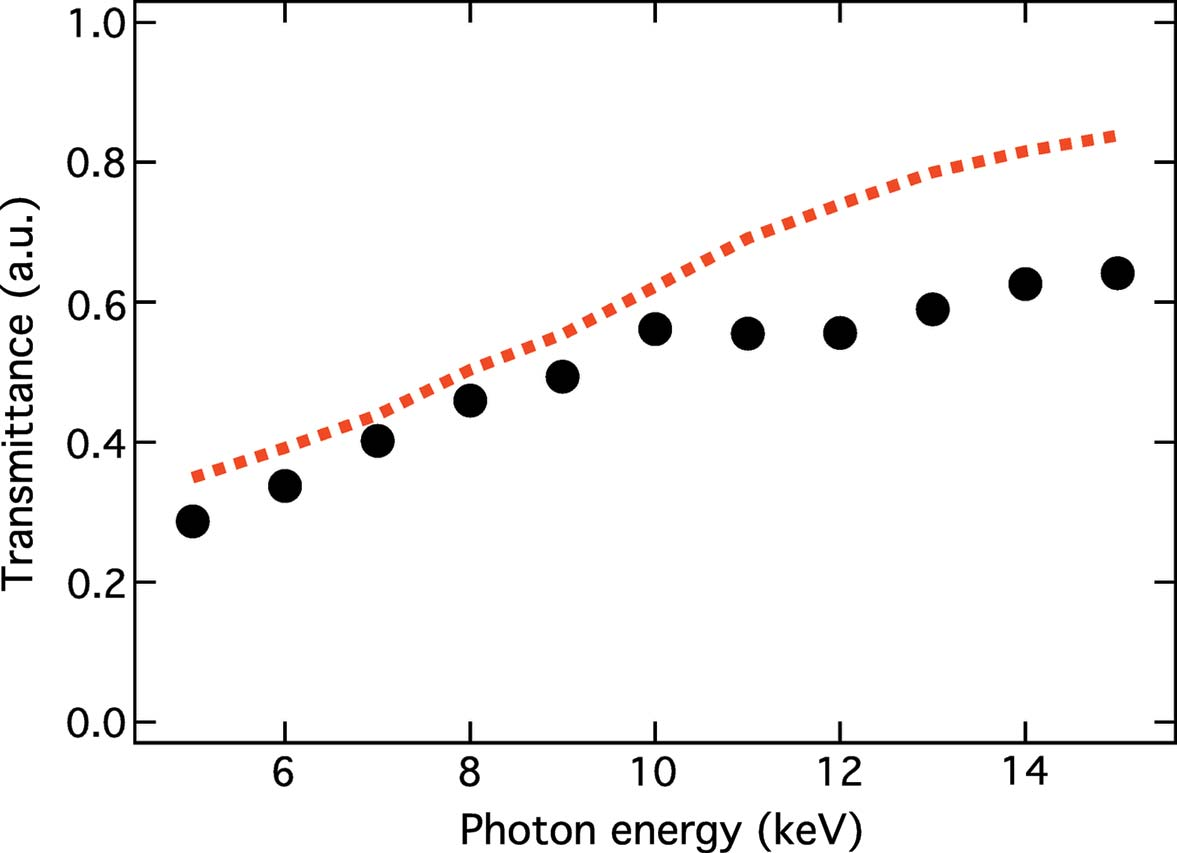
X-ray transmittance of CRLs as a function the photon energy. Black circles are measured data, while the red dotted line corresponds to the calculated values using equation (2).

Assuming a Gaussian beam profile, the X-ray transmittance *T* of the CRLs (Heimann *et al.*, 2016 ▸) is given by
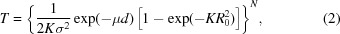
where
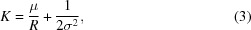
and 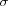, 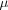, *d* and *N* are the root-mean-square size of the incident beam, the sum of the atomic photoabsorption and inelastic scattering cross sections of beryllium, the space between adjacent paraboloids, and the number of lenses, respectively. Fig. 5 ▸ shows the photon energy dependence of *T*. We observed that the *T* values were slightly smaller than those calculated using equation (2). These deviations may be caused by misalignment to the ideal optical axis, limited stacking precision and surface roughness.

## 4. Application   

**Figure 6:**
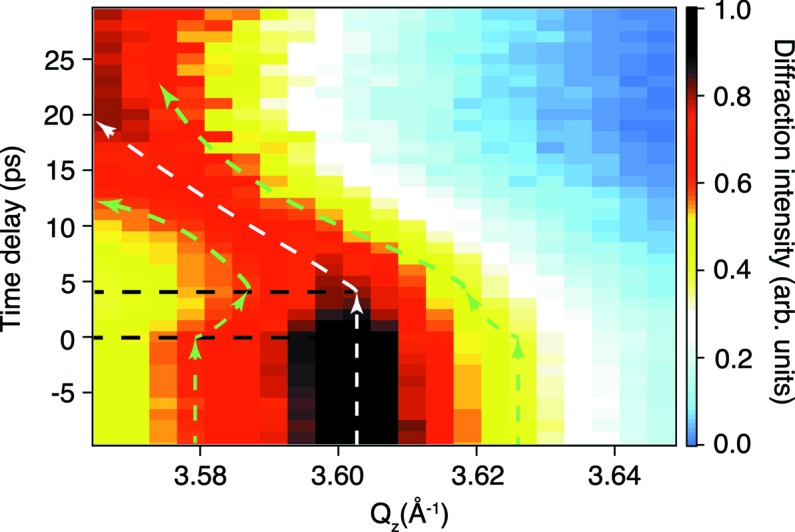
X-ray diffraction rocking curves as a function of time delay for the GST (222) reflection [figure reprinted from Mitrofanov *et al.* (2016 ▸)].

We show a research example to indicate the usefulness of the DCCM and CRLs. Femtosecond X-ray diffraction is one of the most promising tools to directly probe the ultrafast structural change in condensed matter and to elucidate the nature of photoinduced phase transitions. Mitrofanov *et al.* (2016 ▸) measured the (222) diffraction peak of crystalline Ge_2_Sb_2_Te_5_ (GST) thin films, excited with 800 nm optical laser pulses, using the DCCM and CRLs. The time resolution reached 60 fs with the arrival-timing analysis, which corresponds to the time increments used in the time sorting. The experimental data clearly showed that the peak intensity decreased instantaneously after excitation, while the peak position started a shift towards a lower *Q*_*z*_ (corresponding to the lattice expansion) after an ∼4 ps delay (Fig. 6 ▸). The absence of the thermal shift during the first 4 ps indicates non-thermal effects and a metastable intermediate state in photoexcited GST. This intermediate was characterized as a ‘disrupted crystalline’ state; the occupation of anti-bonding states leads to the non-thermal melting of resonant bonds, leaving stronger covalent bonds intact. The remaining short-range-ordered structures can locally relax by tilting or shifting from their original positions. After 4 ps, heating of the lattice occurs via electron–phonon coupling, resulting in a diffraction-peak shift. This study demonstrates the usefulness of the DCCM and CRLs in femtosecond time-resolved X-ray measurements to gain better and deeper understandings of ultrafast photoreactions.

## 5. Conclusions   

We have developed the DCCM and CRLs to tailor the beam conditions of the 0th-order branch. In the performance test, we confirmed that CCs reflected the XFEL beam without unwanted speckles. Focusing of the CRLs achieved minimum spot sizes of ∼1.5 µm × 1.5 µm at the sample position in EH2 for the photon energy range 5–15 keV. These optics will allow users to design pump–probe X-ray experiments with greater flexibilities.

## Figures and Tables

**Table 1:** Parameters of CCs

Type	*A* (high photon energy)	*B* (low photon energy)
Gap (mm)	8	20
Reflection index	Si(111) or Si(333)	Si(111) or Si(333)
Length of L1 (mm)	30	30
Length of L2 (mm)	75	45
Length of L3 (mm)	100	70
Bragg angle (°)	5.78–33.92	20.82–59.93
Photon energy for Si(111) (keV)	3.54–19.63	2.28–5.56
Photon energy for Si(333) (keV)	10.63–58.90	6.85–16.69

**Table 2:** Parameters of CRLs

Photon energy (keV)	5	6	7	8	9	10	11	12	13	14	15
No. of lenses (*R* = 200 µm)	3	4	6	7	10	12	14	17	20	24	27
No. of lenses (*R* = 500 µm)	0	1	0	2	0	1	2	3	3	2	3
